# Supplementary material for: Environmental response in gene expression and DNA methylation reveals factors influencing the adaptive potential of Arabidopsis lyrata
Source: eLife. 2022 Oct 28;11:e83115. doi: 10.7554/eLife.83115 (PMC9616567; doi:10.7554/eLife.83115)
Supplement: Supplementary file 1. [file elife-83115-supp1.docx]

| **ID** | **Field** | **Population** | **Data** | **Number of read pairs (M)** | **Mapping rate** |
| --- | --- | --- | --- | --- | --- |
| 163 | Low | J1 | RNA-Seq | 61.7 | 0.94 |
| 20 | Low | J1 | RNA-Seq | 24.2 | 0.93 |
| 383 | Low | J1 | RNA-Seq | 14.5 | 0.95 |
| 416 | Low | J1 | RNA-Seq | 22.7 | 0.95 |
| 761 | Low | J1 | RNA-Seq | 21.9 | 0.96 |
| 985 | Low | J1 | RNA-Seq | 36.7 | 0.96 |
| 1601 | Low | J3 | RNA-Seq | 21.1 | 0.95 |
| 1651 | Low | J3 | RNA-Seq | 24.5 | 0.95 |
| 1722 | Low | J3 | RNA-Seq | 31.7 | 0.92 |
| 1727 | Low | J3 | RNA-Seq | 21.8 | 0.95 |
| 238 | Low | J3 | RNA-Seq | 47.6 | 0.96 |
| 750 | Low | J3 | RNA-Seq | 42.9 | 0.95 |
| 2187 | Low | GER | RNA-Seq | 38.4 | 0.94 |
| 2196 | Low | GER | RNA-Seq | 46.7 | 0.90 |
| 2214 | Low | GER | RNA-Seq | 24.4 | 0.93 |
| 2260 | Low | GER | RNA-Seq | 24.1 | 0.91 |
| 2304 | Low | GER | RNA-Seq | 30.4 | 0.94 |
| 2324 | Low | GER | RNA-Seq | 13.9 | 0.92 |
| 1159 | High | J1 | RNA-Seq | 29.2 | 0.96 |
| 1490 | High | J1 | RNA-Seq | 26.6 | 0.95 |
| 1492 | High | J1 | RNA-Seq | 30.7 | 0.94 |
| 1658 | High | J1 | RNA-Seq | 28.8 | 0.96 |
| 182 | High | J1 | RNA-Seq | 22.9 | 0.95 |
| 2023 | High | J1 | RNA-Seq | 25.4 | 0.94 |
| 1391 | High | J3 | RNA-Seq | 21.3 | 0.95 |
| 1511 | High | J3 | RNA-Seq | 24.4 | 0.88 |
| 1998 | High | J3 | RNA-Seq | 23.3 | 0.94 |
| 608 | High | J3 | RNA-Seq | 20.8 | 0.95 |
| 762 | High | J3 | RNA-Seq | 23.6 | 0.95 |
| 851 | High | J3 | RNA-Seq | 25.8 | 0.90 |
| 2179 | High | GER | RNA-Seq | 25.6 | 0.93 |
| 2274 | High | GER | RNA-Seq | 31.8 | 0.95 |
| 2285 | High | GER | RNA-Seq | 36.0 | 0.94 |
| 2330 | High | GER | RNA-Seq | 17.6 | 0.92 |
| 2432 | High | GER | RNA-Seq | 18.3 | 0.88 |
| 2456 | High | GER | RNA-Seq | 55.3 | 0.93 |
| 163 | Low | J1 | Bisulfite-Seq | 34.4 | 0.62 |
| 383 | Low | J1 | Bisulfite-Seq | 32.8 | 0.67 |
| 761 | Low | J1 | Bisulfite-Seq | 21.3 | 0.65 |
| 985 | Low | J1 | Bisulfite-Seq | 24.7 | 0.63 |
| 1601 | Low | J3 | Bisulfite-Seq | 25.8 | 0.65 |
| 1651 | Low | J3 | Bisulfite-Seq | 46.9 | 0.66 |
| 238 | Low | J3 | Bisulfite-Seq | 27.4 | 0.65 |
| 750 | Low | J3 | Bisulfite-Seq | 25.4 | 0.64 |
| 2187 | Low | GER | Bisulfite-Seq | 34.0 | 0.65 |
| 2214 | Low | GER | Bisulfite-Seq | 30.2 | 0.65 |
| 2304 | Low | GER | Bisulfite-Seq | 73.8 | 0.60 |
| 2324 | Low | GER | Bisulfite-Seq | 26.4 | 0.66 |
| 1490 | High | J1 | Bisulfite-Seq | 33.0 | 0.64 |
| 1658 | High | J1 | Bisulfite-Seq | 36.4 | 0.65 |
| 182 | High | J1 | Bisulfite-Seq | 24.6 | 0.66 |
| 2023 | High | J1 | Bisulfite-Seq | 30.7 | 0.66 |
| 1123 | High | J3 | Bisulfite-Seq | 38.7 | 0.60 |
| 1222 | High | J3 | Bisulfite-Seq | 33.6 | 0.64 |
| 1528 | High | J3 | Bisulfite-Seq | 33.3 | 0.68 |
| 1817 | High | J3 | Bisulfite-Seq | 29.0 | 0.66 |
| 2274 | High | GER | Bisulfite-Seq | 31.9 | 0.60 |
| 2285 | High | GER | Bisulfite-Seq | 33.3 | 0.58 |
| 2297 | High | GER | Bisulfite-Seq | 16.5 | 0.67 |
| 2456 | High | GER | Bisulfite-Seq | 24.4 | 0.64 |
