# Supplementary material for: Environmental response in gene expression and DNA methylation reveals factors influencing the adaptive potential of Arabidopsis lyrata
Source: eLife. 2022 Oct 28;11:e83115. doi: 10.7554/eLife.83115 (PMC9616567; doi:10.7554/eLife.83115)
Supplement: Supplementary file 2. [file elife-83115-supp2.docx]

| **ID** | **Field** | **Population** | **Sequence context** | | |
| --- | --- | --- | --- | --- | --- |
|  |  |  | **CG** | **CHG** | **CHH** |
| 163 | Low | J1 | 0.997 | 0.997 | 0.998 |
| 383 | Low | J1 | 0.997 | 0.997 | 0.998 |
| 761 | Low | J1 | 0.994 | 0.997 | 0.998 |
| 985 | Low | J1 | 0.998 | 0.997 | 0.998 |
| 1601 | Low | J3 | 0.991 | 0.995 | 0.997 |
| 1651 | Low | J3 | 0.981 | 0.991 | 0.997 |
| 238 | Low | J3 | 0.997 | 0.997 | 0.997 |
| 750 | Low | J3 | 0.997 | 0.997 | 0.997 |
| 2187 | Low | GER | 0.994 | 0.996 | 0.998 |
| 2214 | Low | GER | 0.998 | 0.998 | 0.998 |
| 2304 | Low | GER | 0.997 | 0.997 | 0.998 |
| 2324 | Low | GER | 0.997 | 0.997 | 0.998 |
| 1490 | High | J1 | 0.986 | 0.990 | 0.995 |
| 1658 | High | J1 | 0.995 | 0.996 | 0.997 |
| 182 | High | J1 | 0.997 | 0.997 | 0.997 |
| 2023 | High | J1 | 0.996 | 0.996 | 0.997 |
| 1123 | High | J3 | 0.968 | 0.980 | 0.995 |
| 1222 | High | J3 | 0.937 | 0.967 | 0.993 |
| 1528 | High | J3 | 0.996 | 0.996 | 0.997 |
| 1817 | High | J3 | 0.995 | 0.996 | 0.997 |
| 2274 | High | GER | 0.995 | 0.996 | 0.997 |
| 2285 | High | GER | 0.994 | 0.995 | 0.997 |
| 2297 | High | GER | 0.991 | 0.993 | 0.997 |
| 2456 | High | GER | 0.998 | 0.997 | 0.998 |
