# Supplementary material for: Environmental response in gene expression and DNA methylation reveals factors influencing the adaptive potential of Arabidopsis lyrata
Source: eLife. 2022 Oct 28;11:e83115. doi: 10.7554/eLife.83115 (PMC9616567; doi:10.7554/eLife.83115)
Supplement: Supplementary file 3. [file elife-83115-supp3.docx]

| **Species** | **Source** |
| --- | --- |
| *Arabidopsis halleri* | Ensembl Plants |
| *Arabidopsis thaliana* | Ensembl Plants |
| *Arabis alpina* | http://www.arabis-alpina.org/refseq.html |
| *Boechera stricta* | Phytozome |
| *Brassica rapa* | Ensembl Plants |
| *Camelina sativa* | Ensembl Plants |
| *Capsella rubella* | Phytozome |
| *Cardamine hirsuta* | http://chi.mpipz.mpg.de/assembly.html |
| *Carica papaya* | Phytozome |
| *Citrus clementina* | Ensembl Plants |
| *Cucumis sativus* | Ensembl Plants |
| *Eucalyptus grandis* | Ensembl Plants |
| *Eutrema salsugineum* | Phytozome |
| *Fragaria vesca* | Phytozome |
| *Glycine max* | Ensembl Plants |
| *Juglans regia* | Ensembl Plants |
| *Malus domestica* | Ensembl Plants |
| *Manihot esculenta* | Ensembl Plants |
| *Medicago truncatula* | Ensembl Plants |
| *Pistacia vera* | Ensembl Plants |
| *Populus trichocarpa* | Ensembl Plants |
| *Quercus lobata* | Ensembl Plants |
| *Schenkiella parcula* | Phytozome |
| *Theobroma cacao* | Ensembl Plants |
| *Vitis vinifera* | Ensembl Plants |
